# Genital Schistosomiasis Leading to Ectopic Pregnancy and Subfertility: A Case for Parasitic Evaluation of Gynaecologic Patients in Schistosomiasis Endemic Areas

**DOI:** 10.1155/2013/634264

**Published:** 2013-07-25

**Authors:** Atta Owusu-Bempah, Alexander Tawiah Odoi, Edward Tieru Dassah

**Affiliations:** Department of Obstetrics and Gynaecology, Komfo Anokye Teaching Hospital, P.O. Box KS 1934, Kumasi, Ghana

## Abstract

Female genital schistosomiasis is a significant risk factor for ectopic pregnancy and infertility in schistosomiasis-endemic areas. A case of one previous ectopic pregnancy and subsequent obstruction of the contralateral tube in a secondary subfertility patient with chronic genital schistosomiasis is presented, emphasizing the need for a detailed history and parasitic evaluation of patients presenting with ectopic pregnancy or subfertility in areas where the disease is endemic.

## 1. Introduction

Schistosomiasis affects over 240 million people worldwide, more than 90% of whom live in Africa [1]. Up to 50% of women in *Schistosoma haematobium *endemic areas suffer from genital schistosomiasis. About 23–41% of women with genital schistosomiasis may not excrete any schistosome ova in their urine [2, 3]. Female genital schistosomiasis is associated with significant morbidity including infertility, ectopic pregnancy, and vesicovaginal fistulae [4–9]. A case of one previous ectopic pregnancy and subsequent distal obstruction (hydrosalpinx) of the contralateral tube in a secondary subfertility patient with chronic schistosomiasis is presented. This case highlights two different stages of untreated female genital schistosomiasis in one individual and emphasizes the need for a meticulous history and parasitic evaluation of patients presenting with ectopic pregnancy or subfertility in schistosomiasis endemic areas.

## 2. Case Report

A 34-year-old primiparous woman with a body mass index (BMI) of 22 kg/m^2^ presented to our Gynaecology Clinic with a history of subfertility. She had been married for 10 years prior to her visit and her only child was eight years old. Three years prior to the current visit, she had had an emergency laparotomy on account of tubal ectopic pregnancy and a left total salpingectomy was done; no specimen was sent for histopathological evaluation. The right tube was grossly normal. She had a history of long-standing lower abdominal pain, but no menstrual abnormalities, vaginal discharge, or urinary symptoms such as dysuria, haematuria, or stress incontinence. As a teenager, she used to swim in nearby streams and her past medical history was significant for the treatment of urinary schistosomiasis 19 years prior. She had not had water body (stream, river, dam, pond, or lake) contact since she moved away from her initial location some 14 years prior to this presentation.

Clinical examination, urine and stool examinations, high vaginal and endocervical swabs, and abdominal and pelvic ultrasonography were unremarkable. The husband's semen analysis was normal. However, hysterosalpingography reported a normal intrauterine filling with no defect, right hydrosalpinx, and a left isthmic block (from the previous salpingectomy). The couple was counseled on the diagnosis and the available management options for the subfertility were discussed. The couple agreed to pursue in vitro fertilization and embryo-transfer in the near future. A removal of the right adnexal pathology was advised and the couple consented to elective laparotomy.

Laparotomy revealed a normal-sized uterus, normal looking left ovary and absent left fallopian tube, a hydrosalpinx of the right fallopian tube which was adherent to the right ovary, and a hard mass in the lower aspect of the infundibulo-fimbrial part of the right tube. There was no pus or ascites in the pelvis. A right total salpingectomy with partial resection of the right ovary was done and samples were sent for histopathologic examination. The content of the hydrosalpinx was dark fluid. Her postoperative recovery was uneventful.

**Figure 1:**
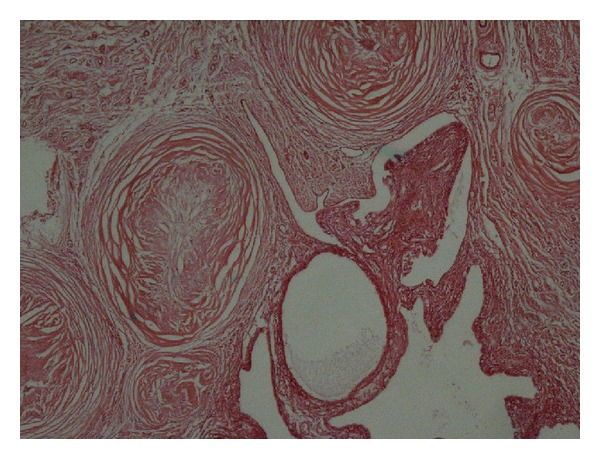
Right fallopian tube showing thickened wall containing many concentric fibroses.

**Figure 2:**
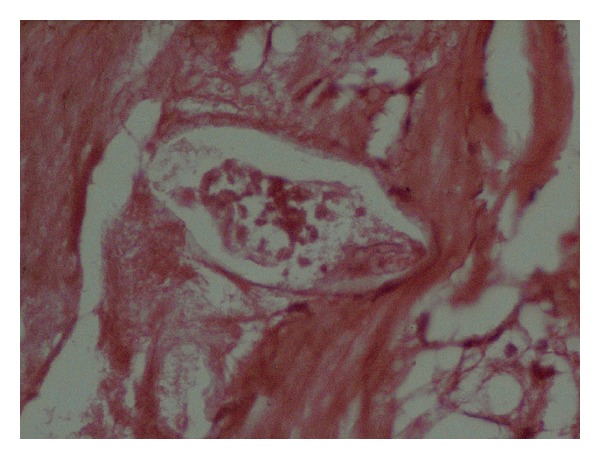
Right fallopian tube showing schistosoma ova in the centre.

Histopathologic evaluation showed cystic dilatation of the fallopian tube with flattening of the epithelium; the wall was thickened, containing many concentric fibroses, some showing granulomas with schistosoma ova in the centre (see Figures 1 and 2). The ovarian tissue was completely replaced by hyalinised, fibrosed concentric nodules and granulomas with schistosoma ova. Admixed chronic inflammatory cells were present at the periphery. A diagnosis of right schistosoma tubo-oophoritis with hydrosalpinx was made.

She was given praziquantel 40 mg/kg body weight daily for three days. The couple was subsequently referred for in vitro fertilization and was hopeful of achieving pregnancy.

## 3. Discussion

Infertility is a major public health problem that affects about 12–30% of couples in sub-Saharan Africa [10, 11]. It has adverse psychosocial and economic implications for affected women/families in most developing countries [11]. In about 85% of women suffering from infertility in Africa, the cause can be attributed to pelvic infections. Although over 70% of these pelvic infections are caused by *Neisseria gonorrhea* and *Chlamydia trachomatis *[10, 11], female genital schistosomiasis remains a significant risk factor for infertility and ectopic pregnancy in schistosomiasis endemic areas where up to 3.6% of ectopic pregnancies and 41% of infertility cases have been attributed to the disease [5, 6].

Genital schistosomiasis can affect all pelvic organs. It is usually asymptomatic but slowly progressive with ova commonly found on the cervix, vagina, ovaries, fallopian tubes, and vulva, and very rarely in the uterus [5, 7, 9]. Manifestations of schistosomal tubal disease span the spectrum of mild reaction to severe fibrotic granulomatous reaction which may impair tubal motility and/or patency, thus predisposing to ectopic pregnancy and infertility [7]. Furthermore, severe perisalpingitis and peritubal adhesions usually result from fallopian tube ischaemia due to ova deposition in the terminal veins of tube [7, 8] as observed in our case.

Although we lack histological data of the left salpingectomy that was done, it is very reasonable to assume that the ectopic pregnancy resulted from tubal schistosomiasis, as the two conditions are known sequelae of untreated schistosomal tubal disease [5, 6, 8]. Our patient gave birth eight years prior to her current visit since her condition was asymptomatic but progressed slowly and the worm(s) continued to lay eggs. It is not clear whether she had been adequately treated for her initial schistosomiasis or she got reinfected. It is rather unfortunate that specimen from the salpingectomy was not sent for histopathological examination, as she would have benefited from antischistosomal treatment and the other tube salvaged from the effects of chronic schistosomiasis (hydrosalpinx). Clearly this case demonstrates two different stages of progressive untreated female genital schistosomiasis in the same patient.

Urine and stool microscopies are the most commonly used methods for the diagnosis of schistosomiasis [5, 6]. However, female genital schistosomiasis poses diagnostic challenges as most patients are either asyymptomatic or do not excrete any ova in their urine or stool [3, 5, 7, 8]. Eosinophilia, abnormal liver, or renal function tests are nonspecific for schistosomiasis and enzyme-linked immunosorbent assays (ELISA) cannot distinguish between active and passive infections [6, 7]. As observed in our case, a detailed history and histopathologic evaluation of relevant specimens (where facilities permit) may be the only practical options in diagnosing genital schistosomiasis in certain situations [3, 7, 8].

In endemic countries, schistosomiasis is usually treated with a single dose of praziquantel (40 mg/kg body weight). However, as this may not result in a complete cure, some authorities recommend treatment for 3 days to ensure complete eradication of the parasite [8]. To improve pregnancy rates following in vitro fertilization (IVF), recent evidence recommends surgical treatment for all women with hydrosalpinges prior to IVF [12]. Since our couple had opted for IVF in the not too distant future, a right total salpingectomy and partial oophorectomy were carried out before referring them for IVF. More importantly, histological examination of the biopsy obtained at surgery confirmed the underlying cause of our patient's tubal pathology.

## 4. Conclusion

Given its gynaecological consequences, the additional morbidity and mortality associated with ectopic pregnancy, and the enormous stigma and socioeconomic ramifications of infertility in most developing countries, female genital schistosomiasis should be of considerable public health importance in endemic countries. Furthermore, considering the diagnostic limitations of urine and stool microscopy, a meticulous history and complete parasitic evaluation (including histological examination of relevant specimen) should be considered an essential component of the management of patients presenting with infertility or ectopic pregnancy in areas where schistosomiasis is endemic.
